# The Challenges of Afghanistan and Iraq Veterans’ Transition from Military to Civilian Life and Approaches to Reconnection

**DOI:** 10.1371/journal.pone.0128599

**Published:** 2015-07-01

**Authors:** Jennifer Ahern, Miranda Worthen, Jackson Masters, Sheri A. Lippman, Emily J. Ozer, Rudolf Moos

**Affiliations:** 1 Division of Epidemiology, University of California, Berkeley School of Public Health, Berkeley, California, United States of America; 2 Department of Health Science and Recreation, San Jose State University, San Jose, California, United States of America; 3 Division of Community Health and Human Development, University of California, Berkeley School of Public Health, Berkeley, California, United States of America; 4 Center for AIDS Prevention Studies, Department of Medicine, University of California San Francisco, San Francisco, California, United States of America; 5 Department of Psychiatry and Behavioral Sciences, Stanford University School of Medicine, Stanford, California, United States of America; 6 Center for Health Care Evaluation, Department of Veterans Affairs Medical Center, Menlo Park, California, United States of America; University of New Mexico, UNITED STATES

## Abstract

Afghanistan and Iraq veterans experienced traumas during deployment, and disrupted connections with friends and family. In this context, it is critical to understand the nature of veterans’ transition to civilian life, the challenges navigated, and approaches to reconnection. We investigated these issues in a qualitative study, framed by homecoming theory, that comprised in-depth interviews with 24 veterans. Using an inductive thematic analysis approach, we developed three overarching themes. **Military as family** explored how many veterans experienced the military environment as a “family” that took care of them and provided structure. **Normal is alien** encompassed many veterans experiences of disconnection from people at home, lack of support from institutions, lack of structure, and loss of purpose upon return to civilian life. **Searching for a new normal** included strategies and supports veterans found to reconnect in the face of these challenges. A veteran who had successfully transitioned and provided support and advice as a peer navigator was frequently discussed as a key resource. A minority of respondents—those who were mistreated by the military system, women veterans, and veterans recovering from substance abuse problems—were less able to access peer support. Other reconnection strategies included becoming an ambassador to the military experience, and knowing transition challenges would ease with time. Results were consistent with and are discussed in the context of homecoming theory and social climate theory. Social support is known to be protective for veterans, but our findings add the nuance of substantial obstacles veterans face in locating and accessing support, due to disconnection and unsupportive institutions. Larger scale work is needed to better understand how to foster peer connection, build reconnection with family, and engage the broader community to understand and support veterans; interventions to support reconnection for veterans should be developed.

## Introduction

United States (US) service members who served in the wars in Afghanistan and Iraq faced long and often multiple deployments and a constant risk of injury and death [1,2]. In addition to exposure to many traumatic events, service members experienced repeated disruption of connections with family members and friends. These disrupted connections, and changes to both the individual and home social environments during separation, lead to a difficult homecoming transition [3–9]. With the formal end of the war in Iraq in late 2011 and a continued disassembling of the large US military presence, veterans are transitioning back to civilian life and it is critical to understand and support their homecoming transition [9].

The high levels of psychological, substance use, and physical health problems in Afghanistan and Iraq veterans are well documented [2,10–13]. Veterans from previous wars who had difficulties in the transition to civilian life faced increased risk of long-term problems that include homelessness, and premature mortality [14–16], indicating that these transition problems are a long-term concern. Given the substantial burden of health problems in recent Afghanistan and Iraq veterans, the potential for long-term impacts is worrisome and support for the transition process is necessary. While research indicates that a successful transition is critical for veterans’ long-term wellbeing, the nature of the transition experience and readjustment needs have not been examined in-depth among Afghanistan and Iraq veterans [17].

Homecoming theory—developed after World War II—serves as a valuable framework for understanding the challenges in the transition from military service [3]. Homecoming theory posits that a traveler such as a military service member is separated from home by space and time. The service member and family members and friends at home have unique experiences during separation. Both the service member and the people and environments at home change during separation, and thus each will be in some ways unknown and unfamiliar to the other upon return. The differences between expectations and reality for the returning veteran and family and friends at home can result in a shock on both sides; navigating homecoming involves reestablishing connections despite these changes.

Homecoming theory emphasizes the theme of disconnection or alienation as part of the challenge faced by returning veterans. Yet little is known about how veterans who served in the wars in Iraq and Afghanistan experience the transition—i.e. the challenges of return to civilian life, key differences between military and civilian environments, how veterans navigate challenges and approach reconnection, as well as what resources mitigate transition difficulties. Gaining insight into the transition experience is critical to understand the difficulties faced by returned veterans as well as to inform interventions to support successful readjustment. To address these issues, we conducted a qualitative investigation of Afghanistan and Iraq veterans’ transition from military to civilian life.

## Materials and Methods

### Recruitment

We conducted in-depth interviews with 24 Afghanistan and Iraq veterans in 2009–2011 in California, US. Veterans were recruited purposively to include both women and men and a range of ages, race/ethnicities, and military service branches. The study was advertised through organizations and events that served a wide array of veterans. Veterans were not recruited based on care seeking or referral for psychiatric or other health problems. Interested veterans contacted the Study Director (MW) and were informed that the study concerned veterans’ experiences upon return from the wars in Iraq and Afghanistan. Veterans were given the option to complete the interview in person or by telephone, and were offered a $50 gift card in compensation for their time. Written informed consent was obtained for in-person interviews, and oral informed consent was obtained for telephone interviews. Written informed consent was not possible for telephone interviews because the respondent and Study Director were in different locations. In instances of oral informed consent, the Study Director printed the respondent’s name, and signed and dated the form to indicate she received consent. All study procedures, including all consent procedures and forms, were reviewed and approved by the Committee for the Protection of Human Subjects at the University of California, Berkeley.

### Interviews

The semi-structured interview guide focused on what was most helpful during the transition to civilian life and what challenges made transition more difficult. Veterans were asked to narrate their experiences, with probes for experiences with family, friends, other veterans, and the general community. See S1 Interview Guide for more details. All interviews were conducted by the Study Director (MW), who has extensive experience conducting qualitative interviews with former combatants. Interviews lasted an average of 60 minutes. Veteran interviews were audiotaped and transcribed.

### Analysis

We applied a thematic analysis approach to the interview data [18,19]. The analysis was primarily inductive, aimed at characterizing key aspects of the transition process by identifying topics that arose repeatedly and considering how those topics were explained by veterans (i.e., what language and metaphors were employed) [18,19]. First, the full data were read and re-read by MW and JA, who separately noted initial ideas about key topics. Then, the data were re-reviewed to identify key ideas that emerged repeatedly and could inform understanding of fundamental aspects of the transition experience (i.e., themes). Interview extracts were classified into these emerging themes. JA refined the themes by checking them against the full data set, reviewed responses that had not fit the dominant patterns, and developed an initial set of overarching and subsidiary themes. JM then reviewed the data and independently coded for the themes developed by JA, and for any additional or modified themes. Subsequently, JA and JM discussed refinements to theme definitions (e.g., **military as family** underwent revision) and added new themes (**unsupportive institutions** was added at this stage). When JA and JM coded different themes, they discussed and came to agreement upon the theme(s) that best fit each interview section. For presentation in this paper, selected quotes are the most evocative and illustrative of a theme, considering also the need for brevity.

## Results

The 24 veterans represented a wide range of demographic and military characteristics (see Table 1). Ages ranged from 22 to 55, 40% were white, and 70% were male. Respondents had served in the Air Force, Army, Marines, Navy, and National Guard and Reserve forces. About 30% had separated from the military in the past year.

**Table 1:** Demographic characteristics of veteran participants.

	N	%
Total	24	100.0
Age		
18–24	4	16.7
25–34	15	62.5
35–44	2	8.3
45–54	2	8.3
55–64	1	4.2
Race/ethnicity		
White	10	41.7
African American	2	8.3
Asian	3	12.5
Hispanic	4	16.7
Unknown	5	20.8
Sex		
Male	17	70.8
Female	7	29.2
Military Branch		
Air Force	2	8.3
Army	8	33.3
Army Reserves	2	8.3
Army National Guard	2	8.3
Army Air Guard	1	4.2
Marines	5	20.8
Navy	4	16.7
Years Since Separation from Military		
<1	7	29.2
1–2	3	12.5
2–3	3	12.5
3–4	5	20.8
4–5	2	8.3
5–6	2	8.3
6–7	1	4.2
Unknown	1	4.2

Three overarching themes were developed and are elaborated below: **military as family, normal is alien,** and **searching for a new normal**.

### 1. Military as family

Many veterans experienced challenging conditions and traumatic events while in the military. These included combat and improvised explosive devices (IEDs), as well as environmental challenges such as sand and wind, and close quarters residence on Naval vessels. Respondents indicated the uniqueness of these experiences with phrases such as “only we know what we’ve been through.” Despite the challenges of service, the military environment itself was experienced as a “family” that took care of service members and provided a structured set of expectations. The overarching theme of military as family contains two subsidiary themes: a) caretaker and b) structure.

#### 1.a. Caretaker

Nearly half (10 of 24) of the veterans viewed the military as an institution that took care of its members. Multiple participants described the military as family, e.g. “the guys that I served with, they were my family over there. So, we have that connection—that bond. And there's always somebody that you can talk to.” Others described the environment in terms reminiscent of the care a family would provide to a child such as “holding their hand,” “safety net,” and “comfort.” One female veteran described how the military fulfilled a parental role related to medical care after she left home:

I went into West Point at 18… [and in the past it was] my parents taking care of all of that, and then [the military] taking care of all that… as someone who had major medical work done and was going to need more done it was frightening. I didn’t understand how [civilian health care] worked, and honestly I barely understand how it works now… I was used to being in a system where everything just made sense.

A career Reservist who had a leadership position described taking on a parental role in helping calm distressed soldiers in challenging situations by giving them concrete and simple tasks to complete.

A small minority of veterans thought that the military had turned against them during service. They expressed a profound feeling of betrayal, analogous to the experience of being betrayed by family. As one female veteran who had been sexually assaulted in the military explained, “I mean, Iraq—you expect people to shoot at you, you expect people to die, you expect people to be killed. You don't expect your fellow soldiers to turn on you.”

#### 1.b. Structure

Nearly half of veterans (11 of 24) noted that the structure in the military environment provided clarity and simplicity to decisions and procedures. They described the structure of the system as something to “hold onto” in the chaos of a war zone, and also as a framework that provided the opportunity to excel. The environment was described as “black and white”—a setting in which orders are given and obeyed. A male veteran in his mid 20s described the structure as a key reason that some people return to the military after separation, “they want that structured life again because… you get used to it if you do it long enough. And then you get out, then all of a sudden you've got to take care of yourself.” This description also illustrates how structure is an important component of military family caretaking. Similarly, the career Reservist who gave soldiers concrete and simple tasks in stressful situations was providing structure as a way to take care of his soldiers.

### 2. Normal is Alien

The overarching theme of **normal is alien** captures veterans’ experience of alienation upon return to civilian life. Veterans frequently talked about civilian life as “normal” while it was clear that many aspects of civilian life no longer felt normal to them upon return from military service. This reflected the mismatch between expectations that coming home would be a welcome return to “normal”, and the reality that what used to feel “normal” felt alien due to the changes in the veteran and changes at home. This overarching theme is directly connected to **military as family**, which contains the subsidiary themes of caretaker and structure, because the departure from the structure and caretaking within the military environment was a key part of what generated a feeling of alienation from “normal” civilian life. **Normal is alien** includes four subsidiary themes: a) disconnection, b) unsupportive institutions c) lack of civilian structure and d) loss of purpose.

#### 2.a. Disconnection

Upon return to civilian life the vast majority veterans (19 of 24) felt disconnection from people at home, including family and friends, who had not shared the experience of military service. Veterans felt that those who had not served in the war could not truly understand them or their experiences during service. As one respondent lamented, “I can tell stories all night long and [my family] probably won’t really grasp what’s going on.” Most families were making major efforts to provide support to veterans, but due to this feeling of disconnection, veterans faced difficulties in accessing the support offered. Veterans also described feelings of disconnection when friends, acquaintances and strangers made “unwarranted assumptions” about people in the military and military service, asked insensitive questions about veterans’ experiences in service (e.g., “did you kill anyone?”), or tried to connect veterans experiences to their own in a way that was not seen as respectful.

#### 2.b. Unsupportive institutions

Veterans expected their service to be honored, but many (15 of 24) felt they did not receive deserved support from the military, the Department of Veterans Affairs (VA), and other institutions in the transition back to civilian life. This generated and exacerbated feelings of alienation. Some veterans reported that the military did not provide needed resources for the transition to civilian life. As one respondent explained, “A lot of the [Officers] don't send people to [the class to facilitate transition to civilian life]. And the class is available. It's not like they don't offer it.”

Other respondents reported that their mental health problems were not appropriately diagnosed or handled. As a veteran who had recently retired from a long military career explained, “All too often, they refer to these injuries as the invisible injuries of warfare… They're not invisible to loved ones… So who are they invisible to?… They're invisible to leaders who don't want to recognize the mental and emotional and spiritual sides of warriors.” Others reported that the medical care provided by the VA was inadequate.

Beyond the VA, some veterans reported inflexibility of community institutions. For example, one respondent explained the frustrations of being unable to translate military emergency medical technician (EMT) training and experience into a civilian EMT job, “‘You can drive an ambulance… [but] you can’t treat anybody.’ But I’ve done all these things. Look at my training certificate. ‘Oh we don’t honor that certificate.’ That’s really hard to deal with.” Another veteran described being unable to take advantage of a veteran loan program because the banks he approached did not want to deal with the restrictions and costs.

#### 2.c. Lack of civilian structure

A quarter of respondents (6 of 24) expressed that the difference between the highly structured military environment and the less structured civilian environment created challenges in organizing their lives, and frustration in dealing with people at home. For example, one respondent said “… everything [in the military] is structured…. And then you get out and all of a sudden you've got to take care of yourself.” Another veteran talked about this issue as one of the biggest challenges, “…just switching from that military style of chain of command and clear goal into all of a sudden having to make your own decisions. I think a lot of guys have trouble with that.” This problem also manifested in frustration with family members, friends and classmates (for veterans who attended college) when they were not punctual and did not respect authority. One respondent noted:

…there are certain expectations that you can rely on while you're over there, such as… [when] things need to be done, they're done, [because] your life depends upon it. Whereas here, there's a lot more leeway. And initially when I came back, I just couldn't deal with that gray area that's neither black nor white, and people talking back [and] making excuses… So I was pretty quick to get mad or frustrated.

#### 2.d. Loss of purpose

A substantial proportion of respondents (10 of 24) noted that civilian life lacked meaning and purpose, and that they no longer felt they were contributing to an important communal effort. As a female veteran in her mid 50s explained:

It’s really hard to put in words but I just miss the environment. I miss the common goals… the way people put aside their own personal [agendas]… I used to run a lot and [the] feeling is just like that…‘yeah man, let’s go do it!’ I’ve never really gotten the same thing on the civilian side, even though I try and pour my heart into things.

The experience of a lack of meaning was intensified when veterans could not find jobs they felt were important or drew upon their skills. As a young male veteran reflected:

When I did start working at the surplus store… I had a very mixed feelings about it… I saw myself doing security [or] first aid, something that validated my experiences over there. But then after searching for a job for two months, I was grateful to have found one. So, it was such a mixed feeling and I guess a wake-up call, you know, that it just didn't matter that I had been to Iraq.

### 3. Searching for a new normal

The overarching theme of **searching for a new normal** captures the approaches, resources and perspectives that helped support veterans’ successful transition to civilian life. For many veterans, family members were an important foundation that offered practical help such as a place to live, and tried to understand and support them. However, veterans often found it hard to engage that support due to feelings of alienation from individuals who had not shared the experience of military service. Three subsidiary themes emerged about connections or perspectives that eased the challenges of transition and supported veterans to find their way to a fulfilling civilian life: a) support from a navigator, b) embracing an ambassador role, and c) ease with time.

#### 3.a. Support from a navigator

Veterans who had help from a veteran peer or a veteran specific support system to navigate the transition to civilian life had substantial advantages. For 13 of 24 this was an important source of support. Veteran peers provided invaluable help with practical issues such as how to access veteran benefits, and how to manage more general civilian tasks. As one veteran explained, “What’s been helpful, really, is just friends [who] have gone out before me. They’ve been really helpful in telling me… what needs to be done after the military. They had nobody to really guide them, they had to find out the hard way.” A key feature was that these veterans had successfully navigated the transition out of the military; peers who were themselves in the throes of transition challenges were not consistently perceived as helpful or supportive. Another veteran found that an uncle who was also a veteran was the only one who was able to continue to support him as he struggled with a drug addiction, “He would call, and he would always let me know that he'd be there, and whenever I was ready to come back to earth, he would be there. But my mom and everybody, they just didn't--no one understood what I was going through.”

Veterans coordinators at colleges and universities also provided important support and practical help, “There was [a veterans representative at the University]… She was always asking me how I was doing in my classes, and then she pointed me in the right direction [to get services from] the VA.” While most of the comments about navigators focused on help provided with practical tasks, it was clear that emotional support was also conferred as part of the help provided by navigators.

In contrast, veterans who had been mistreated or betrayed while in the military, such as those who experienced sexual assault, often did not feel comfortable with those who served in the military. Thus they could not access veteran peers as a source of support and had to contend with psychological effects of the mistreatment, as well as alienation from people at home. Women veterans were less able to connect to other veterans, although they did find veterans coordinators at colleges and universities to provide helpful support. A few veterans recovering from substance use problems found that spending time with veteran peers was not supportive of recovery because alcohol use was common, and thus they also faced challenges in accessing peer support.

#### 3.b. Embracing an ambassador role

Some veterans (7 of 24) found that embracing the role of being an ambassador to the military experience was a way to connect to people at home and to feel like they were doing something important. One veteran explained how he tried to help civilians understand his experience in the service, “they think ‘Saving Private Ryan’. And that’s not true… so every time I talk to a civilian I have to explain. It’s like giving a lesson.” In addition to explaining the military experience to civilians, some veterans were asked to describe their experiences at public events, “Memorial Day they asked me to wear my uniform and say a couple of things. So I did, and it was fun… I got a lot of handshakes. It was cool.” This approach helped mitigate feelings of alienation.

#### 3.c. Ease with time

Some veterans (8 of 24) discussed the challenges of transition as inevitable but easing over time. The initial months of transition were often characterized by considerable alienation, more frequent substance use, and conflicts with others. As one veteran described, “I know I always offend people. And then I just say ‘Oh, I’m sorry. It’s only been 90 days. It’s the crazy period.’ And then I always calm down afterward.” Simply knowing that the challenges of transition would ease with time helped veterans be patient with the experience. Overall, veterans who discussed this topic indicated that about a year was required to readjust to civilian life. Veterans who described struggles lasting well beyond a year were generally dealing with serious exacerbating issues such as psychopathology, substance use problems, or experiences of sexual assault in the military.

## Discussion

### Summary

Overall, a substantial proportion of veterans experienced the military as a family that took care of them and provided structure. Upon return to civilian life, many veterans experienced alienation and lack of support from institutions—including from the military and veterans’ agencies themselves—and faced a substantial lack of structure and loss of purpose. In the face of these challenges, the most common resource for a successful transition back to civilian life was a veteran who had successfully transitioned, and provided support and advice as a peer navigator. A peer who has successfully adapted to civilian life may uniquely understand another veteran’s experiences and be able to provide timely help navigating benefits (e.g., GI Bill, VA) and other practicalities. A minority of respondents—those who reported the military system had turned against them, women veterans, and veterans recovering from substance abuse problems—were less able to access peer support.

### Experiences in the military and “military as family”

Consistent with homecoming theory, many veterans reported experiences during military service that they viewed as unique, including combat and environmental challenges [3]. These shared experiences strengthened their connection to others who served due to the potential for mutual understanding, and created disconnection from friends and family upon return from service, also consistent with the theory. Social climate theory posits that support, goal/task orientation, and structure/organization are three key underlying characteristics of many institutional and group contexts, and our findings that the military provides caretaking, purpose, and structure are congruent with this well-established and empirically supported theoretical framework [20].

### Homecoming in which “normal is alien”

The experience of alienation described by many participants is consistent with homecoming theory, which posits that environmental differences and personal change lead to a mismatch between expectations and reality when the veteran returns; this experience has also been documented in research on US veterans of previous wars [3–7]. Recent work has described this phenomenon in Afghanistan and Iraq veterans as being “out of sync” due to military and civilian cultural difference [21], and feeling like they do not “fit with society” [17]. Feelings of alienation increase the likelihood of risky behaviors beyond the effect of PTSD symptoms, indicating that they have a distinct role in hampering reintegration and wellbeing [22].

Problems with unsupportive institutions have also emerged in work with college student veterans, who described “feeling abandoned” when they did not receive helpful transition training from the military, and when they faced difficulties accessing VA services [17]. Lack of support from institutions generates and exacerbates alienation. Some veterans felt they could not connect with family, friends, and other civilians, and, to make matters worse, institutions that should have provided support left them feeing mistreated and unappreciated. Awareness of institutional problems that veterans face, such as those documented related to the VA, may lead to service improvements [23]. The Institute of Medicine’s recommendation for structural changes to create dedicated time and space for decompression and transition training programs for returning military may also improve transition experiences for veterans [24].

The struggles veterans experience with a lack of structure and purpose upon return to civilian life are key emerging issues in research in Afghanistan and Iraq veterans. Differences between military and civilian culture have increased over the past century, and issues of structure and purpose are exacerbated in an environment in which it is difficult to find employment [25]. Problems faced with lack of structure are similar to discussions of lack of control and discipline in civilian life as a challenge for returning World War II soldiers [3]. However, that work emphasized the frustrations veterans had with other people, while our findings highlight challenges for veterans in organizing their own lives and finding a sense of meaning without military structure and goals. Consistent with our findings on structure, other research identified this as an important challenge for veterans in transition and suggested that spending time with others with military background could be helpful [26]. However, this strategy might be more difficult for veterans who find connecting with other veterans more challenging, such as those who reported the military system had turned against them. Consistent with our findings on loss of purpose, recent work with Afghanistan and Iraq veterans revealed feelings of uselessness and a lack of importance in work and school endeavors [21].

While the challenges faced by returning veterans are in many ways unique, homecoming theory postulates certain commonalities of homecoming transitions [3]. Consistent with this theory, our findings have parallels with research on other return transitions, including prisoners reentering society, returning refugees and return migrants. For example, following release, prisoners face challenges with a loss of structure, lack of institutional support, and difficulties navigating family roles [27]. Similar challenges are experienced by returning refugees and return migrants [28–30].

### Searching to reconnect and find “a new normal”

Homecoming theory posits that reestablishing connections is critical to a successful transition [3]. Although social support has been related to lower risk of mental health and related problems among veterans [31–41], our research brings a nuanced perspective to those findings, highlighting that many veterans face substantial obstacles in navigating to a situation in which they are supported. Many veterans felt alienated from family and friends, and mistreated by institutions that might have provided support. In this setting, alternative sources of support that can serve as a link from old to new and foster reconnection at home are important during transition. While family members and other civilians’ attempts to understand their experiences and provide support were often perceived as ineffective, the support veterans provided to one another was more often effective. The findings of this study indicate that peer support may be a critical part of successful transition. Larger scale study of the kinds of support veterans provide to one another, the timing of support received, and how support affects reintegration can shed light on how the process of support develops and could potentially inform peer support intervention.

Other work with veterans has recommended fostering relationships with military peers as a way to support reintegration, but the focus has been primarily on emotional support rather than navigational and practical support [26]. Interventions to connect veterans to support transition navigation merit consideration; they have been piloted for linkage to mental health care, but there are limited data on effectiveness, and potential challenges include some individual’s reluctance to participate and concern that a formal program may not realize the same benefits as support that develops through natural peer connections [34,42–45]. Future research should examine acceptability and potential effectiveness of a formal broad peer program for transition navigation (i.e., not focused only on linkage to mental health care) to help inform intervention efforts. Development of any peer support program should consider the important minority of veterans who may face challenges with veteran peers, and might be able to connect better with peers who have shared experiences (e.g., connect transitioning veterans recovering from substance abuse with veterans in recovery) or might need completely distinct non-peer sources of support. Also, veterans who experience alienation from family are of major concern and efforts to support reconnection are needed. Approaches to rebuild connection with family members, such as mindfulness and relaxation massage based interventions, may be effective and should be considered for broader implementation [46].

Some veterans were able to take on the role of an ambassador as a way to connect with civilians, and it is possible that embracing an ambassador role might be helpful for more veterans. Reaching out in this way to bridge the divide required a degree of stability and confidence, but also might help build a sense of confidence that could assist veterans in making a healthy transition. Overall, the findings indicate that veterans are helping one another, and helping civilians to understand them. It would be worth exploring how civilians could do more to effectively bridge the gap, and to reduce the perception that individuals who have not been in service cannot understand veterans’ experiences. Although the experiences at war may be difficult to understand, most civilians have experienced the feeling of alienation and could empathize with that aspect of the experience. In addition, there are many civilians who have made transitions between cultural contexts during their lives who would understand issues of separation, change and disconnection. The notable success of some representatives at colleges in helping veterans indicates that with the right training, support and connection is possible. There are high quality materials and trainings on what the community can do to support veterans, such as those developed by Swords to Plowshares [47], and it would be worth finding ways that key messages could be more broadly disseminated.

### Strengths and limitations

To our knowledge, this is the first study of a sample drawn from a general population of veterans to examine the transition experience in depth; a recent study examined the transition experience of seven veterans in college [17]. A major strength is that our qualitative approach enabled us to identify themes that characterized the transition experience; these methods are well suited to capturing phenomena and patterns in veterans’ transition experiences. We recruited participants through a wide array of veteran contacts and networks, and did not recruit from any health care seeking context. This assures that our veteran sample includes a diverse set of backgrounds and does not over represent care-seeking veterans. Although it is possible that we were less likely to successfully recruit veterans who faced more difficulties in transition, we do note that several participants who had gone through extremely difficult transitions did share their experiences with us. This analysis reflects the themes that arose in this small and diverse veteran sample, and thus while the themes represent common experiences across a wide range of veteran backgrounds, it is important to emphasize that our design, sampling approach, and sample size were not suited nor intended to enable broad generalizable claims regarding the overall population of veterans.

## Conclusion

Overall, it is critically important to better support veterans in transition given the long-term risks to those who do not transition successfully [14,15,48]. Our research suggests that supports that veterans provide one another are extremely helpful in navigating a successful transition, and it is thus well worth conducting larger scale work to better understand how to foster peer connection, and to develop interventions informed by this work. Further research needs to consider how to support the subgroup of veterans who may face challenges connecting with veteran peers. Given the pervasive feeling of alienation expressed by many returning veterans, it is also important to develop ways to foster reconnection between veterans and their families, and to explore means to engage the broader community in understanding and supporting returning veterans.

## Supporting Information

S1 Interview Guide In-depth Interview Questions for Veteran Interviews. (PDF)
