# Supplementary material for: The Challenges of Afghanistan and Iraq Veterans’ Transition from Military to Civilian Life and Approaches to Reconnection
Source: PLoS One. 2015 Jul 1;10(7):e0128599. doi: 10.1371/journal.pone.0128599 (PMC4489090; doi:10.1371/journal.pone.0128599)
Supplement: S1 Interview Guide — (PDF) [file pone.0128599.s001.pdf]

IDI Questions: Veteran Pilot

- What has been helpful or made things easier during your transition out of the military? *(e.g. People who have been supportive? Activities you have enjoyed? Work? School?)*
- What has made things more difficult?

- What people or groups do you feel most comfortable spending time with? Why? *(e.g. family, friends, other Veterans, community members, by yourself?)*
- Are there any particular places where you feel most comfortable, or activities that you find enjoyable or helpful? Why? *(places: e.g., outdoors, at home, at school, at work. activities: e.g., exercise, watching TV, reading)*
- How have things been with your family since you returned? *(Do you feel supported by your family? Is this a change since you were deployed/in the military?)*
- How have things been with your friends? *(Do you feel supported by your friends? Is this a change since you were deployed/in the military?)*
- How have things been in your community where you live or your neighborhood? Do you feel connected to your neighborhood? Do you think people in your neighborhood can understand or relate to what you experienced in the military? *(Is this a change since you were deployed/in the military?)*
- What is your impression of how people in your neighborhood feel about Veterans and about the conflicts in Iraq and Afghanistan? (*e.g., ever stigmatized by being a Veteran or involved in the wars? ever given better treatment by being a Veteran or involved in the wars?*)
- Do you feel part of a community of Veterans? *(if yes, please describe your Veteran community; if no, do you experience any challenges to being part of a Veteran community?)*

- What do you think are the biggest problems that OEF/OIF Veterans face when they return to civilian life?

- Do you think some OEF/OIF Veterans have trouble related to drinking after they leave the military? Do you think some Veterans use tranquilizers (*like Quaalude (kway-lood), Seconal (sek-uh-nawl) or "reds", Valium, Xanax (zan-ax), Librium (lib-ree-um), or Ativan*)? What about other drugs? *(If yes to any, why do you think Veterans have trouble with drinking/other drugs?)*

Basic Demographic Info:

- Age
- Rank
- Military Branch
- Where deployed
- Dates of deployment/separation
- Member of any Veteran organizations?
